# Malignant Transformation in Extraoral Lichen Planus: A Systematic Review and Meta-Analysis in the Context of the Risk in Oral Lichen Planus

**DOI:** 10.3390/dj14040217

**Published:** 2026-04-08

**Authors:** Ana Carolina Evangelista Colafemina, Caique Mariano Pedroso, Maria Eduarda Perez-de-Oliveira, Ana Gabriela Costa Normando, Katherine France, Rafael Tomaz Gomes, Marcelo Henrique Napimoga, Marcio Ajudarte Lopes, Alan Roger Santos-Silva

**Affiliations:** 1 Oral Diagnosis Department, Piracicaba Dental School, University of Campinas (UNICAMP), Piracicaba 13414-903, Brazil; anacarolina.colafemina@gmail.com (A.C.E.C.); gabinormando@gmail.com (A.G.C.N.); malopes@fop.unicamp.br (M.A.L.); 2 Department of Clinical and Preventive Dentistry, Universidade Federal de Pernambuco, Recife 50670-420, Brazil; meduardaperezo@gmail.com; 3 Department of Oral Medicine, School of Dental Medicine, University of Pennsylvania, Philadelphia, PA 19104, USA; kfrance@upenn.edu; 4 Private Practice, Sao Paulo 01000-000, Brazil; 5 Faculdade São Leopoldo Mandic, Instituto de Pesquisas São Leopoldo Mandic, Campinas 13045-755, Brazil; marcelo.napimoga@slmandic.edu.br

**Keywords:** lichen planus, genital, cutaneous, malignant transformation, systematic review

## Abstract

**Objectives**: This systematic review aimed to evaluate malignant transformation (MT) in extraoral lichen planus to help contextualize the debated malignant potential of oral lichen planus in a mucocutaneous disease model. **Methods**: A comprehensive literature search was conducted across five databases and gray literature sources, without date restrictions. Observational studies reporting MT in cutaneous or genital LP were included. Data extraction, risk of bias assessment using Joanna Briggs Institute (JBI), and meta-analysis of proportions were performed. Subgroup analyses by anatomical site were conducted where possible. **Results**: Ten studies (15,829 patients) were included, with a predominance of women (93.1%). The pooled MT rate of extraoral LP was 1% (95% CI: 0.00–0.03). Subgroup analysis revealed a significantly higher rate in vulvar LP (2%; 95% CI: 0.02–0.03) compared to cutaneous LP (0%; 95% CI: 0.00–0.01) (*p* < 0.0001). Squamous cell carcinoma was the most frequent malignancy. The erosive and hypertrophic subtypes of LP were more commonly associated with cancer development. **Conclusions**: MT of extraoral LP appears to be rare, particularly in cutaneous forms. However, the risks observed in the genital mucosa reinforce the need for accurate diagnosis and long-term follow-up, especially in erosive presentations.

## 1. Introduction

Lichen planus (LP) is a chronic inflammatory autoimmune disease that affects the skin, scalp, nails, and mucosal surfaces, particularly the oral and genital regions [1]. Although its etiology remains unclear, LP is more prevalent in women, who may also be more susceptible to psychological and physical factors that can influence disease onset and progression [2].

LP occurs in several clinical forms, most commonly reticular, erosive, and atrophic. The erosive and atrophic forms are usually symptomatic and require corticosteroid therapy and stress management, while asymptomatic cases benefit from regular follow-up [3,4,5]. Cutaneous LP typically involves flexor surfaces, presenting as violaceous, symmetrical papules. Diagnosis is mainly clinical but is often confirmed by histopathology [4,6].

The malignant transformation (MT) potential of LP remains a matter of ongoing debate. While the World Health Organization (WHO) and international consensus documents have classified oral LP as a potentially malignant disorder, reported MT rates in oral LP vary widely across studies, and concerns persist regarding diagnostic misclassification, particularly the overlap between oral LP and oral lichenoid lesions, as well as the inconsistent inclusion or exclusion of epithelial dysplasia at baseline [7]. These issues complicate causal inference and continue to fuel debate regarding whether oral LP should be regarded as a potentially malignant disorder, or whether the observed malignant outcomes partially reflect heterogeneous case definitions and inclusion of lesions with intrinsic dysplastic risk.

As LP is a mucocutaneous disorder, the biological plausibility of MT, if truly linked to LP-related chronic inflammation, would not be expected to be confined exclusively to the oral cavity. Proposed mechanisms include chronic inflammation-induced oxidative stress, activation of oncogenic pathways, and the release of free radicals and growth factors [8,9,10]. If LP intrinsically confers malignant potential, a measurable risk signal might also be detectable in other anatomic subsets where LP occurs, especially in chronically inflamed mucosa (e.g., genital or esophageal sites). Conversely, if extraoral LP shows negligible transformation rates, this would provide contextual evidence relevant to the ongoing debate regarding whether oral LP should indeed be considered a potentially malignant disorder, or whether observed oral risks may be more strongly influenced by confounding factors and diagnostic overlap.

A previous systematic review assessed the malignant potential of vulvar LP and lichen sclerosus [11]. However, it did not evaluate other extraoral sites, such as cutaneous, esophageal, or anal LP. Therefore, given the debated malignant potential of oral LP and the mucocutaneous nature of the disease, the present systematic review aimed to synthesize the available evidence on malignant transformation in extraoral LP. The review addressed the question “What is the proportion of malignant transformation in cutaneous and extraoral mucosal LP?” and explored whether transformation rates differ by anatomic site, thereby contributing to a broader understanding of whether LP, particularly oral LP, should be considered potentially malignant.

## 2. Materials and Methods

### 2.1. Eligibility Criteria

The inclusion criteria were defined using the PECOS framework (Population, Exposure, Comparison, Outcome, and Study design), which guided both the formulation of the review’s focused question and the eligibility criteria: (P) Patients diagnosed with extraoral LP, including cutaneous, genital, esophageal, or other mucosal sites beyond the oral cavity. No restrictions regarding age, sex, race, or geographic region. Diagnosis of LP had to be confirmed clinically and/or histopathologically. (E) Presence of extraoral LP. (C) Not applicable. (O) Prevalence of MT in cutaneous and extraoral mucosal LP. (S) Cohort and case–control studies.

Exclusion criteria were as follows: (1) studies that did not assess or report data on MT associated with extraoral LP (e.g., those focusing exclusively on oral LP or unrelated dermatological conditions); (2) studies including malignant lesions that were not associated with, or not preceded by, a confirmed diagnosis of extraoral LP; (3) studies lacking extractable or site-specific data for extraoral LP (e.g., grouped data for oral and extraoral LP without stratification); (4) reviews, protocols, short communications, personal opinions, letters, case reports, case series, conference abstracts, and experimental research; (5) studies whose full texts were not available; (6) studies published in languages other than English, Spanish, or Portuguese.

This systematic review was conducted in accordance with the PRISMA 2020 guidelines, and the corresponding checklist (File S1) is provided as Supplementary Material.

### 2.2. Information Sources and Search Strategy

References were identified through tailored search strategies conducted on 26 May 2025, across the following electronic databases: Scopus, PubMed/MEDLINE, Web of Science, LILACS, and Embase. In addition, a gray literature search was performed using Google Scholar and ProQuest. The reference lists of the included studies were also manually screened to identify any additional relevant publications. All retrieved records were imported into the EndNote Web (Clarivate Analytics, Philadelphia, PA, USA), where duplicated references were removed. No restrictions on publication date were applied in the search strategy. The detailed search strategies are provided in Supplementary Table S1.

Detailed information regarding the search strategy is available in Supplementary Table S1. The search strategy was intended to capture all extraoral sites of lichen planus, including esophageal, genital, and cutaneous locations. Specific descriptors were primarily applied to improve the retrieval of studies focused on cutaneous and genital involvement.

### 2.3. Selection Process

After reference identification, the selection process was conducted in two phases. In Phase 1, titles and abstracts were independently screened by two reviewers (A.C.E.C. and C.M.P) using the online software Rayyan^®^ (Qatar Computing Research Institute, Doha, Qatar) [12]. In Phase 2, the same reviewers assessed the full text to determine the final eligibility. The reasons for exclusion at this stage were recorded and used to construct the article selection flow chart (Supplementary Table S2). Divergences in both phases were initially resolved through discussion between the two reviewers. When consensus could not be reached, a third author (M.E.P.O.) was consulted to make the final decision.

### 2.4. Data Collection Process and Data Items

Data extraction was performed by one reviewer and independently verified by a second reviewer to ensure accuracy. The following information was extracted from each included study: study characteristics (author, year of publication, country, and study design), population characteristics (sample size, patients’ sex, age, and comorbidities, diagnostic methods for LP, lesion location, clinical signs and symptoms, follow-up duration, and treatment), and MT data (cancer diagnosis, time interval between LP diagnosis and cancer development, clinicopathological features of the malignancy, cancer treatment, patient outcomes). Both qualitative and quantitative data were tabulated using Microsoft Excel^®^.

### 2.5. Risk of Bias Assessment

The risk of bias for individual studies was independently evaluated by two reviewers using the Joanna Briggs Institute (JBI) Critical Appraisal Tools specific to each study design [13,14]. Studies were classified as having a high risk of bias if the proportion of “yes” responses was up to 49%, moderate risk if between 50% and 69%, and low risk if at least 70%. Disagreements were resolved by discussion, and a third author was consulted in cases where divergences remained.

### 2.6. Effect Measures

The primary outcome of this systematic review was the proportion of MT among cases of extraoral LP. The occurrence of MT was reported using absolute and relative frequencies with 95% confidence intervals (CI).

### 2.7. Synthesis of Results

A meta-analysis of the MT rate in extraoral LP was conducted using RStudio (Version 4.3.1, Boston, MA, USA), with the meta-package. A proportion meta-analysis was performed using the inverse variance method and Freeman-Tukey Double arcsine transformation to stabilize variances and pool prevalence estimates. Statistical heterogeneity was calculated using an inconsistency index (I^2^) and Cochran’s Q test. A random-effects model with a 95% CI was used to generalize the findings beyond the included studies. Subgroup analysis was conducted to compare vulvar and cutaneous LP.

## 3. Results

### 3.1. Search and Study Selection

**Figure 1:**
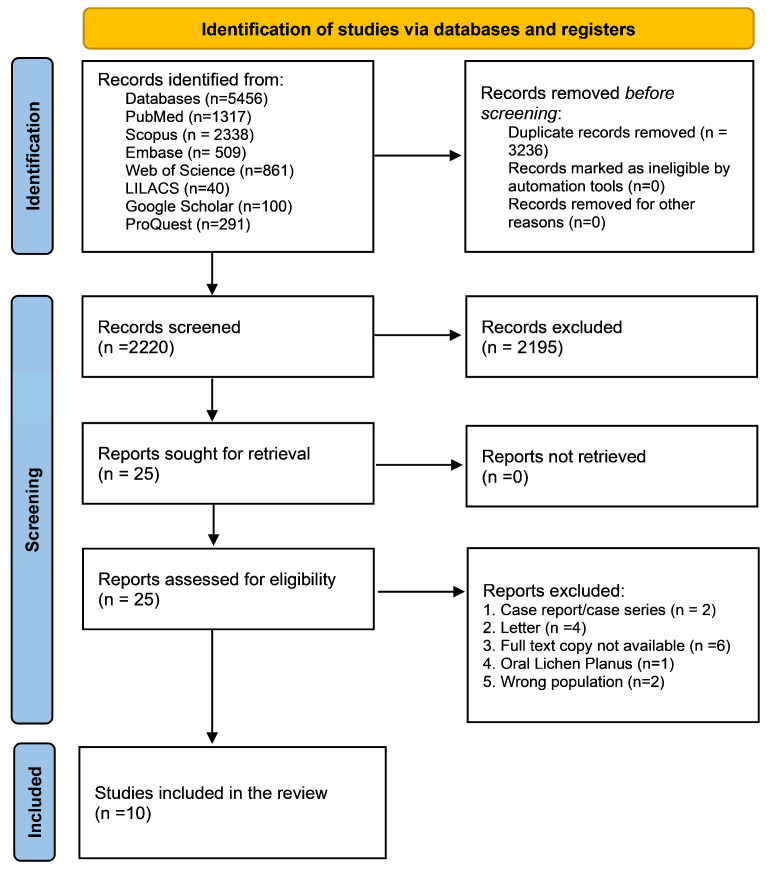
PRISMA flow diagram of the study selection process. From 5456 identified records, 10 studies were included in the qualitative synthesis and 8 in the quantitative analysis. Reasons for exclusion at each stage are detailed in the diagram. Adapted from Page et al., 2020 [15].

The literature search retrieved a total of 5065 references from five electronic databases. After the removal of duplicates, 3078 records were screened by title and abstract. Nineteen articles were selected for full-text reading. An additional 391 records were identified through gray literature searches, from which five more articles were included. In total, 10 articles met the inclusion criteria, with eight eligible for quantitative analysis. Two studies were not included in the quantitative synthesis because their designs were not compatible with the meta-analysis of proportion. One study used a case–control design, and another included only patients who had already developed malignant transformation, without reporting the total number of patients with lichen planus at risk (Figure 1).

### 3.2. Study Characteristics

Among the 10 included studies, one was a case–control study and 9 were cohort studies. The studies were published between 1991 and 2025, and were conducted in eight different countries: Finland (2) [16,17], the Netherlands (2) [18,19], the United States (1) [20], Portugal (1) [21], Austria (1) [22], England (1) [23], Sweden (1) [24], and Germany (1) [25]. Detailed characteristics of the included studies are presented in Supplementary Table S3.

The total sample comprised 15,829 patients, with a predominance of female participants (14,772 cases; 93.1%). The mean age was 54.1 years, ranging from 1–96 years. Most studies focused on vulvovaginal involvement, although some also reported extra-genital manifestations, including cutaneous, esophageal, and ocular locations. Comorbidities were inconsistently reported; among the five studies that mentioned them, the most frequent were autoimmune diseases, obesity, contact allergies, and endocrine/metabolic disease (Table 1).

Signs and symptoms were reported in six studies. The most frequent symptoms at presentation included pruritus, pain or burning sensation, dyspareunia, and structural anatomical alterations. Other symptoms included dryness, irritation, dysuria, and vaginal discharge. Erosive LP was typically associated with severe discomfort and mucosal fragility, while hypertrophic LP presented as thickened, hyperkeratotic plaques, often in the lower extremities (Table 1).

The erosive subtype was most common in cases involving the genital region. Other variants included hypertrophic LP, particularly associated with cutaneous SCC, and classic LP. Some studies did not specify the subtype but confirmed the diagnosis histologically. Others used registry data without a confirmatory biopsy (Supplementary Table S3).

Diagnosis was established clinically and confirmed by histopathological examination in most studies. Among those with histological confirmation, common features included a band-like lymphohistiocytic infiltrate. However, some studies included patients based solely on clinical criteria, and in one study, the diagnostic method was based solely on registry data (Supplementary Table S3).

Topical corticosteroids were the first-line treatment across nearly all studies, used in most patients. Clobetasol propionate (0.05%) was the most prescribed agent. Systemic therapy was prescribed in four studies, including drugs such as oral corticosteroids, hydroxychloroquine, methotrexate, acitretin, and cyclosporine. Some regimens also incorporated calcineurin inhibitors, and in one case, intravenous immunoglobulin. Surgical management was indicated in selected cases to correct adhesions, introital narrowing, or anatomical distortion caused by chronic inflammation (Supplementary Table S3 and Table 1).

### 3.3. Risk of Bias

**Figure 2:**
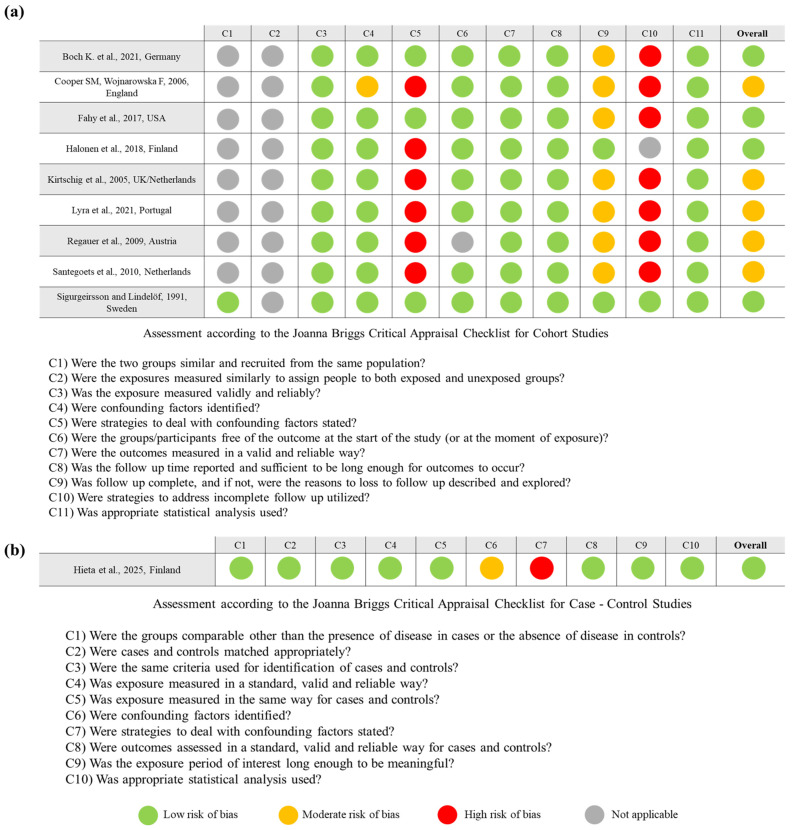
Risk of bias assessment of included studies using the JBI Critical Appraisal Checklist [13,14]. The overall classification was based on the proportion of criteria met. (**a**) Assessment of cohort studies [16,18,19,20,21,22,23,24,25]. (**b**) Assessment of the case–control study [17].

Four cohort studies demonstrated low risk, while the remaining five were judged to have a moderate risk of bias (Figure 2a). The main limitations observed among the moderate-risk studies included a lack of strategies to address confounding factors (C5), incomplete or insufficiently justified information regarding participant attrition (C9), and inadequate reporting or duration of follow-up (C10). Specifically, the studies by Cooper and Wojnawoska (2006) [23], Kirtschig et al. (2005) [18], Lyra et al. (2021) [21], and Regauer et al. (2009) [22] showed high risk in domains C5 and C10, indicating potential bias related to confounding and attrition. The study by Santegoets et al. (2010) [19] also lacked clarity in reporting confounding and follow-up, while that by Halonen et al. (2018) [16] had moderate concerns regarding participant inclusion (C4) and reporting (C9).

The single case–control study was classified as showing a low risk of bias (Figure 2b), although minor concerns were noted regarding confounding (C6 and C7), which were not fully addressed.

### 3.4. Results of Individual Studies

Across the included studies, a total of 100 cases of MT were reported. The genital region, particularly the vulva, was the most common site (70 cases, including 68 vulvar and 2 penile), followed by the esophagus (19 cases), skin, and larynx/epiglottis. The average time to cancer diagnosis was 26.6 months for genital LP and 178 months for cutaneous LP (Table 2).

Among patients who developed cancer, the erosive and hypertrophic subtypes were the most associated with malignancy in the genital region, with 22 (46.8%) and 25 (53.2%) cases, respectively (Supplementary Table S3 and Table 2). In contrast, cutaneous transformation was more commonly observed in patients with hypertrophic LP. The classic subtype was less frequent and reported in only one study (Supplementary Table S3 and Table 2).

Histologically, squamous cell carcinoma (SCC) was the malignancy associated with LP across the included studies. Among the studies that specified the histological subtype of carcinoma, 3 patients were diagnosed with carcinoma in situ, 1 patient with invasive carcinoma, and 1 patient with stage 1A. Additionally, two studies reported cases of high-grade squamous intraepithelial lesions (HSIL) without evidence of progression to invasive carcinoma. Notably, one study specified that tumors were HPV- and p16^INK4a^ negative, supporting the hypothesis that LP-related carcinogenesis may follow an HPV-independent pathway (Supplementary Table S3).

Surgical excision was the primary treatment modality for LP-associated carcinomas, reported in four studies, and employed for approximately 45 patients. This included partial or radical vulvectomy in genital cases, and excisional surgery with lymphadenectomy when indicated. Chemoradiotherapy was employed in 2 patients. In 6 studies, the treatment regimen for cancer was not disclosed (Supplementary Table S3 and Table 2).

Only two studies provided follow-up information. While one of them reported favorable clinical outcomes without recurrence or metastatic progression, the other study highlighted a high mortality rate associated with LP-associated SCC, which was attributed to the aggressive nature of these tumors, frequent regional lymph node metastases at diagnosis, delayed recognition of malignancy, and residual diseased mucosa that contributed to early recurrences (Supplementary Table S3 and Table 2).

### 3.5. Results of Synthesis

**Figure 3:**
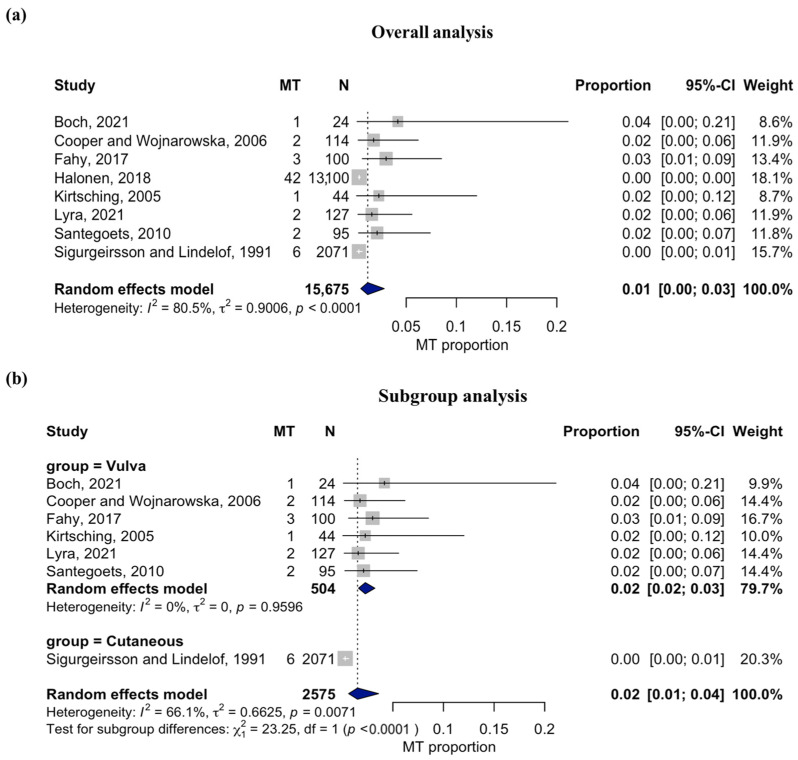
(**a**) Forest plot of the overall MT rate of extraoral LP. The pooled proportion was 1% (95% CI: 0.00–0.03), based on 8 cohort studies including 15,675 patients. Heterogeneity was substantial (I^2^ = 80.5%, *p* < 0.0001) [16,18,19,20,21,23,24,25]. (**b**) Subgroup analysis of MT rates according to anatomical site. The pooled MT rate was 2% (95% CI: 0.02–0.03) for vulvar LP and 0% (95% CI: 0.00–0.01) for cutaneous LP. The difference between subgroups was statistically significant (*p* < 0.0001) [18,19,20,21,23,24,25]. Squares represent the effect size of each study, with their size proportional to the study weight. Horizontal lines indicate 95% confidence intervals. The diamond at the bottom represents the pooled estimate with its corresponding confidence interval.

The pooled MT rate was 1% (95% CI: 0.00–0.03), with heterogeneity among studies (I^2^ = 80.5%; *p* < 0.0001) (Figure 3a). To explore potential sources of heterogeneity, a subgroup analysis based on anatomical location was performed. The vulvar subgroup, which included six studies with a combined total of 504 patients, showed a pooled transformation rate of 2% (95% CI: 0.02–0.03) with no observed heterogeneity (I^2^ = 0%). In contrast, the only study focused on cutaneous LP (*n* = 2071) reported a MT rate of 0% (95% CI: 0.00–0.01), with moderate heterogeneity (I^2^ = 66.1%; *p* = 0.0071). The difference between subgroups was statistically significant (*p* < 0.0001) (Figure 3b).

Of the eight cohort studies included in the overall meta-analysis, one could not be incorporated into the subgroup analysis because it did not distinguish the anatomical sites of LP involvement.

## 4. Discussion

This review found a low overall MT rate in extraoral LP, with a pooled prevalence of 1%, consistent with a previous systematic review reporting a rate of 1.6% in vulvar LP [11]. Notably, the genital location appears to be one of the most affected regions in extraoral LP, a pattern also observed in the current review [26,27]. The prevalence of women and the involvement of genital and limb areas suggest a possible predilection for specific anatomical sites. Our subgroup analysis further supports this observation, showing a higher transformation rate of 2% in the vulvar region compared to 0% in cutaneous LP. These results suggest that mucosal involvement, particularly in the vulva, may be more prone to MT than cutaneous or other extraoral sites.

Among the different clinical variants, erosive LP was most frequently observed, followed by hypertrophic LP [27]. The erosive subtype, often symptomatic and associated with chronic inflammation, has long been associated with a higher risk of cancer development [28]. This aligns with our results and supports the hypothesis that persistent mucosal inflammation contributes to oncogenic progression [29]. From an immunopathogenic perspective, LP is characterized by a cytotoxic T-cell–mediated autoimmune response against antigens presented by dendritic cells and keratinocytes [1,2]. Keratinocyte apoptosis is mainly induced by activated CD8+ T cells through mechanisms such as FAS–FAS ligand interactions, granzyme B release facilitated by perforin-mediated membrane pores, and tumor necrosis factor-alpha (TNF-α) signaling [1,2,30]. In addition, alterations in cytokine profiles have been reported across clinical subtypes, including increased interferon-gamma (IFN-γ) levels and variations in interleukin-4 (IL-4) expression, particularly in erosive and erythematous/ulcerative forms of LP [31,32,33,34].

In contrast, MT of cutaneous LP remains rare and controversial, as also reflected in our review. Reported risks vary, from approximately 0.3% in vulvar LP to around 6% in esophageal LP, although larger and more robust studies are needed to confirm these estimates [35,36]. Notably, in our dataset, two studies described around 25 patients with esophageal LP, and an additional study reported 19 patients who developed MT at this site. Although these observations are based on very limited evidence, they highlight the need for further investigation.

The role of chronic inflammation in the pathogenesis of cancer in LP has been widely discussed. Persistent inflammation releases cytokines and growth factors that drive mutations and cellular changes, potentially leading to MT [37]. This microenvironment, characterized by the presence of activated immune cells and a complex network of cytokines, may facilitate the development of SCC. Macrophages, mast cells, fibroblasts, and T lymphocytes secrete factors such as TNF, macrophage migration inhibitory factor (MIF), matrix metalloproteinases (MMPs), chymase, and IL-4/IL-6, promoting angiogenesis, extracellular matrix remodeling, and degradation. These events affect oral epithelial cells, driving abnormal growth, apoptosis resistance, increased motility, and neoplastic transformation [38]. In addition, chronic ultraviolet (UV) exposure has been proposed as a contributing factor, particularly in cases of cutaneous LP. Krasowska et al. (2012) reported that cancer development in LP lesions may have been influenced by UV radiation [39]. Given that UV exposure is a well-established risk factor for non-melanoma skin cancer, it is plausible that, in sun-exposed areas, UV radiation may represent a greater risk for MT than LP itself [40].

Although chronic exposure to UV radiation has been suggested as a risk factor for MT in cutaneous LP, it is important to highlight that phototherapy, particularly narrowband UVB (NB-UVB), has been used as a therapeutic approach in some cases. Studies by Fernández-Guarino et al. and Pavlotsky et al. reported response rates between 70% and 80%, with prolonged remission in many patients [37,41]. These beneficial effects are attributed to immunomodulatory effects, including T-cell apoptosis and reduction in inflammatory cytokines. None of the included studies reported phototherapy use in LP cases that developed malignancy, limiting conclusions about its impact on cancer risk.

Extraoral LP commonly affects the wrists, ankles, trunk, and genital region, which is consistent with our findings [26,27]. The malignant potential of LP in the oral site has been debated for years. The first case of LP-related malignancy was reported in 1910 [42]. In addition, a systematic review carried out by Giuliani et al. (2019) showed a potential for malignancy of OLP of 1.4% [43]. Likewise, some authors do not report any cancer progression [44]. In 2003, the WHO revised its criteria to differentiate LP from lichenoid lesions due to the significant diagnostic overlap. Lichenoid reactions, microscopically, may present epithelial dysplasia, which justifies the higher observed rates of MT [45]. As a result, the misdiagnosis may contribute to the variability in reported transformation rates.

Our systematic review found some limitations across the included studies. One study only enrolled patients who had already developed carcinomas, which may have inflated the perceived risk [22]. In another report, the diagnosis of LP was based solely on clinical examination, without histopathological confirmation, which may introduce a risk of misclassification [24]. In some studies, not all patients had histopathological confirmation of LP, which raises concerns about diagnostic accuracy and the potential inclusion of clinically similar but distinct conditions [18,19,21,23]. Importantly, the diagnosis of LP in clinical practice typically relies on clinicopathological correlation rather than exclusively clinical or histopathological criteria. Histopathology alone may not reliably distinguish LP from other lichenoid conditions, such as lichenoid drug reactions or vulvar lichen sclerosus, which may contribute to diagnostic heterogeneity across studies. In addition, potential confounding factors for SCC, including UV exposure, smoking, immunosuppression, and HPV infection, were inconsistently reported across studies, limiting causal interpretation. Furthermore, only one study focused on cutaneous LP [24], and no eligible studies addressed esophageal, nail, or other sites, limiting broader conclusions about these less common presentations. Moreover, due to incomplete reporting, it was not possible to stratify all outcomes by LP subtype or anatomical site, limiting the scope of our subgroup analyses.

Although the prevalence of MT could be estimated, it was not feasible to conduct a formal risk analysis, such as calculating odds ratios, to assess causality. This was mainly due to the lack of studies specifically designed to evaluate risk, the absence of control groups, inconsistent follow-up information, and heterogeneous study designs. These methodological constraints limit our ability to determine whether LP independently increases the risk of malignancy compared to unaffected populations. These gaps reinforce the need for prospective studies with standardized diagnostics and long-term follow-up. Reducing heterogeneity will improve accuracy and reporting, while insights into the LP immune microenvironment are already driving safer and more effective therapies.

These findings also help contextualize the debate regarding the malignant potential of oral LP. As LP is a mucocutaneous disorder, a carcinogenic potential would be expected to manifest across different anatomical sites affected by the disease. However, the low rate of malignant transformation observed in extraoral LP suggests that transformation is uncommon outside the oral cavity. This observation provides additional context for interpreting the risk attributed to oral LP. It reinforces the possibility that some of the variability reported in studies on oral LP may be influenced by diagnostic challenges, particularly the overlap with lichenoid lesions and the inclusion of lesions with baseline epithelial dysplasia.

Overall, MT in extraoral LP appears uncommon, even in mucosal sites such as the genital region. Mucosal tissues outside the oral cavity share similar histopathological and inflammatory characteristics, which reinforces the need for careful differential diagnosis from other lichenoid disorders. Continued research using consistent diagnostic criteria will be essential to clarify the true malignant potential across different clinical variants and anatomical locations.

## 5. Conclusions

This systematic review found that extraoral LP has a low rate of MT, with a pooled estimate of 1%. The vulvar region demonstrated a relatively higher occurrence of malignant progression, whereas cutaneous involvement was associated with fewer cases. Although some instances of transformation were documented, the available evidence is still too limited to confirm a definitive association.

## 6. Other Information

This systematic review was performed according to the Preferred Reporting Items for Systematic Reviews and Meta-Analyses statement guidelines (PRISMA) [15]. The protocol was registered at the International Prospective Register of Systematic Reviews (PROSPERO) under registration number CRD42022319289 [46].

## Figures and Tables

**Table 1:** Characteristics of studies on lichen planus.

	Number of Patients *
Sex	
Female	14,746
Male	1083
Age (mean)	56.8
LP site	
Cutaneous	2139
Genital mucosa	658
Oral	157
Oropharyngeal	47
Perianal	45
Esophagus	25
Scalp	20
Nail	4
Ocular	2
Comorbidities	
Postmenopausal	92
Autoimmune diseases	56
Obesity	37
Prior childbirth	31
Allergic/Atopic	30
Endocrine/Metabolic	23
Smoking	17
Cardiovascular	17
Cancer	7
Asthma	2
Psychiatric	1
LP type	
Erosive	313
Hypertrophic	31
Classic	9
Treatment	
Topical corticosteroids	390
Systemic corticosteroids	93
Surgery	43
Signs and Symptoms	
Pain, burning sensation, pruritus	604
Dyspareunia	245
Structural anatomical alterations	146
Erosion	125
White reticulation	94
Dryness	62
Irritation	55
Erythema	51
Dysuria	44
Glazed erythema	44

Footnote: * The numbers reported for LP sites, clinical types, comorbidities, treatments, and signs/symptoms correspond to cases in which these variables were specifically reported in the included studies. These categories are not mutually exclusive, and therefore the totals do not necessarily add up to the overall number of patients with LP.

**Table 2:** Characteristics of malignant transformation on LP.

	Cancer Diagnosis	Time from Diagnosis of LP to Cancer (m)	Type of LP			Treatment			Patient Status
			Erosive	Hypertrophic	Classic	Surgical	Chemotherapy	PDT	
Genital	70	26	19 (46.8%)	25 (53.2%)	0	45 (93.7%)	2 (4.2%)	1 (2.1%)	Alive (26)/Dead (14)
Esophagus	19	N/R	N/R	N/R	N/R	N/R	N/R	N/R	N/R
Cutaneous	6	178	0	4 (66.7%)	2 (33.3%)	N/R	N/R	N/R	N/R
Larynx/epiglottis	5	N/R	N/R	N/R	N/R	N/R	N/R	N/R	N/R

Footnote: Not reported (N/R).

## Data Availability

The data supporting this systematic review were obtained from previously published sources, which are fully referenced in the article. The complete data extraction and analysis spreadsheet is available from the corresponding author upon reasonable request. We hereby confirm that this manuscript, or any part of it, has not been submitted or published elsewhere and will not be submitted for publication in another journal. Analyses were conducted in R Studio (Version 4.3.1) using the meta package for meta-analysis. Custom R scripts were developed to perform the proportion meta-analysis, calculate heterogeneity statistics (I^2^ and Cochran’s Q), and generate subgroup analyses. The code supporting the findings of this article is available from the corresponding author upon reasonable request.

## Supplementary Materials

The following supporting information can be downloaded at: https://www.mdpi.com/article/10.3390/dj14040217/s1, Table S1: Table of Search Strategies in Databases, Table S2: Excluded articles and the reasons for their exclusion, Table S3: Summary of descriptive characteristics of the 10 included studies regarding LP malignancy, File S1: PRISMA 2020 Checklist.

## Abbreviations

The following abbreviations are used in this manuscript:

MT	Malignant transformation
SCC	Squamous cell carcinoma
LP	Lichen planus
PRISMA	Preferred Reporting Items for Systematic Reviews and Meta-Analyses statement guidelines
WHO	World Health Organization
JBI	Joanna Briggs Institute
