# Supplementary material for: Malignant Transformation in Extraoral Lichen Planus: A Systematic Review and Meta-Analysis in the Context of the Risk in Oral Lichen Planus
Source: Dent J (Basel). 2026 Apr 8;14(4):217. doi: 10.3390/dj14040217 (PMC13114415; doi:10.3390/dj14040217)
Supplement: Supplementary file 1 [file dentistry-14-00217-s001.zip › dentistry-4160844-supplementary-Table S3.pdf]

Supplementary Table S3. Summary of descriptive characteristics of the 10 included studies regarding LP malignancy

Author, year, country	Type of study	Sample size / sex	Age (mean/range)	Comorbidities	LP type	Techniques used for diagnosis	Signs and symptoms	Affected sites	Treatment	Follow-up (m)	MT	Cancer type	Type of malignant LP	Follow-up until cancer diagnosis (m)	Cancer treatment	Patient status
**Boch K. et al., 2021, Germany**	Retrospective cohort	24 F	60.9 years<br>**Range:** 30-85	Hypertension: 12 (50%)<br>Diabetes mellitus: 5 (20.8%)<br>Hypothyroidism: 5 (20.8%)<br>Hyperlipidemia: 4 (16.7%)<br>Asthma: 2 (8.3%)<br>Ischemic heart disease: 1 (4.2%)<br>Depression: 1 (4.2%)	Erosive: 15 (62.5%)<br>Classic: 7 (29.2%)<br>Hypertrophic: 2 (8.3%)	Clinical + Histopathological (band-like lymphohistiocytic infiltrate and basal degeneration)	Glazed erythema: 20 (83.3%)<br>Scarring lesions: 15 (62.5%)<br>Erosions 14 (58.3%)<br>Burning sensation: 10 (41.7%)<br>Hyperkeratotic borders: 2 (8.3%)	Genital: 24 (100%)<br>Cutaneous: 7 (29.2%)<br>Oral: 6 (25%)	**Topical treatment**: 24 (100.0%) - potent and/or super-potent topical corticosteroids, pimecrolimus/tacrolimus<br>**Systemic treatment**: 12 (50.0%) - acitretin, corticosteroids, or hydroxychloroquine<br>**Surgical treatment**: 5 (20.8%) - adhesions and scarring	19.3	1 (4.2%)	Vulvar SCC, Stage 1A	Erosive	17	N/R	N/R
**Cooper SM, Wojnarowska F, 2006, England**	Prospective cohort	114 F	61.2 years<br>**Range**: 27 - 82	N/R	Erosive	Clinical diagnosis<br>Histopathological: 97 (85%)	Erosion: 111 (97%)<br>Pain/soreness: 91 (80%)<br>White reticulation: 94 (82%)<br>Clitoral burying: 77 (68%)<br>Pruritus: 74 (65%)<br>Dyspareunia: 70 (61%)<br>Introital narrowing: 67 (59%)<br>Irritation: 55 (48%)<br>Erythema: 51 (45%)<br>Dryness: 31 (27%)<br>Hyperkeratosis: 25 (22%)<br>Pallor: 24 (21%)<br>Glazed erythema: 24 (21%)<br>Fissures: 2 (2%)<br>Purpura: 1 (1%)<br>Telangiectasia: 1 (1%)	Genital: 114 (100%)<br>Perianal: 32 (28%)<br>Oral: 67 (59%)<br>Cutaneous: 22 (19%)<br>Scalp: 9 (8%)<br>Nails: 4 (4%)<br>Esophagus: 2 (2%)	**Topical preparations**: 89 (78%) - 0.05% clobetasol propionate, tacrolimus<br>**Systemic treatments:** 31 (27.2%) - minocycline hydrochloride, erythromycin ethyl succinate, combined minocycline hydrochloride and niacinamide, prednisolone, acitretin, cyclosporine, azathioprine sodium, hydroxychloroquine sulfate, thalidomide, colchicine.<br>**Surgical treatment:** 21 (18%) - correction of introital stenosis and division of vulval/vaginal adhesions	72	2 (2%)	Vulvar SCC	Erosive	N/R	N/R	N/R
**Fahy et al., 2017, USA**	Retrospective cohort	100 F	60.3 years<br>**Range:** 27 - 89	Autoimmune disease: 22% (22)<br>Thyroid disease: 17% (17)<br>Vitiligo: 1% (1)<br>Rheumatoid arthritis: 1% (1)<br>Celiac disease: 1% (1)	N/R	Clinical + Histopathological (band-like infiltrate at the dermoepidermal junction and lichenoid mucositis)	Dyspareunia: 91 (91%)<br>Pain, burning sensation, pruritus: 69 (69%)<br>Burning sensation, pruritus: 8 (8%)<br>Pain: 7 (7%)<br>Pruritus: 4 (4%)<br>Pain, pruritus: 2 (2%)	Genital: 100 (100%)<br>Oropharyngeal: 47 (47%)<br>Cutaneous: 20 (20%)<br>Scalp: 11 (11%)<br>Esophagus: 4 (4%)<br>Ocular: 2 (2%)	**Topical**: 47 (47%) – corticosteroids, clobetasol, tacrolimus, pimecrolimus<br>**Systemic**: 43 (43%) - Hydroxychloroquine, methotrexate, mycophenolate mofetil, oral corticosteroids<br>**Others**: Intravenous IgG 2 (4.7%), antibiotics/antifungals 30 (0%)	39.5	3 (3%)	Vulvar SCC *in situ:* 1<br>Invasive vulval SCC: 2	N/R	N/R	N/R	N/R
**Halonen et al., 2018, Finland**	Cohort study (population-based register data)	13,100 F	N/R	N/R	N/R	Diagnoses extracted from Finnish Hospital Discharge Registry (ICD codes)<br>Clinical or Histopathological confirmation not specified (registry limitation)	N/R	Not specified individually, but LP sites include cutaneous, oral cavity, genital, esophagus, larynx, pharynx	N/R	N/R	Esophag us: 19<br>Larynx/ epiglottis : 5<br>Vulva: 18	N/R	N/R	N/R	N/R	N/R
**Hieta et al., 2025, Finland**	Case- control	60 M<br>56 F	F: 59.9 years<br>**Range:** 24 - 88<br>M: 40.8 years<br>**Range:** 8 - 91	N/R	N/R	Histopathological	N/R	Genital: 116 (100%)	N/R	N/R	F:1<br>M:2	Vulvar SCC *in situ:* 1<br>High-grade HSIL: 2	N/R	45.6	Surgery: 3<br>PDT: 1	N/R

												Penile SCC *in situ:* 1<br>Penile SCC: 1				
**Kirtschig et al., 2005, UK/Netherlands**	Retrospective cohort	44 F	57 years<br>**Range:** 23 - 87	Prior childbirth: 31 (70%)<br>Smoking: 12 (27%)<br>Thyroid disease: 7 (16%)<br>Cancer: 7 (16%) – one vulvar<br>Diabetes mellitus: 2 (5%)<br>Psoriasis: 2 (5%)<br>Vitiligo: 1 (2%)	Erosive	Clinical + Histopathological (38/44 patients)	Soreness: 33 (75%)<br>Pruritus: 28 (64%)<br>Dryness: 31 (27%)<br>Dyspareunia: 22 (50%)<br>Vaginal Bleeding: 11 (25%)<br>Fusion of the vagina: 2 (4.5%)	Genital: 44 (100%)<br>Oral: 30 (68%)<br>Cutaneous: 9 (20%)	**Topical**: 44 (100%) – 0.05% clobetasol propionate, 0.025% beclometasone dipropionate	N/R	1 (2.3%)	Vulvar SCC	Erosive	18	N/R	N/R
**Lyra et al., 2021, Portugal**	Retrospective cohort	127 F	59 years<br>**Range:** 17 - 90	Obesity: 37/83 (45.1%)<br>Smoking: 5/122 (4.1%),<br>Postmenopausal: 92/123 (74.8%),<br>Hormone therapy: 11/83 (13.3%)	Erosive	Clinical: 108/127<br>Histopathological: 19/127	Vulvar itching: 91 (77.8%)<br>Burning: 52 (44.4%)<br>Dysuria: 37 (31.6%)<br>Perianal itching: 22 (22%)<br>Vulvar pain: 20 (17.7%)<br>Vaginal discharge: 14 (12.1%)	Genital: 127 (100%)<br>Perianal: 13/127 (10.4%)	**Topical:** 122 (91.8%) clobetasol propionate, 32 (25.2%) hydrocortisone, betamethasone, and triamcinolone. 10 cases (8.2%) calcineurin inhibitors<br>**Systemic:** 1 (0.8%) steroid, 4 cases (3.3%) methotrexate, 2 cases (1.6%) retinoids<br>**Other:** 1 (0.8%) - Fourchette plasty	46.8	2 (1.6%)	Vulval SCC: 2<br>HSIL: 1	Erosive	45	Surgery	No recurrence, metastasis, or disease-related deaths
**Regauer et al., 2009, Austria**	Retrospective cohort	38 F	61 years<br>**Range:** 39-90	N/R	Erosive: 13<br>Hypertrophic: 25	Clinical diagnosis + Histopathological<br>HPV DNA testing (INNO-LiPA)<br>Immunohistochemistry for p16^{INK4a} and p53	N/R	Genital: 38 (100%)	N/R	57	38 (100%)	Vulvar SCC	Erosive: 13<br>Hypertrophic: 25	N/R	Surgery: 38 (100%)<br>Chemoradiotherapy: 2 (5.2%)	Fourteen of 38 (37%) patients died of SCCs
**Santegoets et al., 2010, Netherlands**	Retrospective clinical	95 F	55 years<br>**Range:** 24 - 80	Contact allergies: 16 (16.8%)<br>Atopic disease: 14 (14.7%)<br>Thyroid disease: 3 (3.1%)<br>Vitiligo: 1 (1.05%)	N/R	Clinical + Histopathological (81/95 - 85.3%)	Dyspareunia: 51 (53.7%)<br>Vulval soreness and burning: 47 (9.5%)<br>Vulval pruritus: 46 (48.4%)<br>Contact bleeding: 16 (16.8%)<br>Easy bleeding gums: 13 (13.7%)<br>Inability to engage in intercourse: 11 (11.6%)<br>Vaginal discharge: 8 (8.4%)<br>Dysuria: 7 (7.4%)	Genital: 95 (100%)<br>Oral: 54 (56.84%)<br>Cutaneous: 10 (10.5%)	**Topical:** 64 (67.4%) clobetasol propionate 0.05%; 27 (28.4%) fluticasone propionate 0.005%; 4 (4.2%) intravaginal 10.0% hydrocortisone acetate<br>**Surgery:** 17 (17.9%)	N/R	2 (2.1%)	Vulvar SCC	N/R	N/R	Surgery	N/R
**Sigurgeirsson and Lindelöf, 1991, Sweden**	Retrospective cohort	1048 F<br>1023 M	52 years<br>**Range:** 1–96	N/R	Classic and Hypertrophic	Clinical	N/R	Cutaneous: 2071 (100%)<br>Oral: unspecified	N/R	118.8	6 (0.2%)	SCC	Hypertrophic: 4/6<br>Classic: 2/6	178	N/R	N/R
