# Supplementary material for: Malignant Transformation in Extraoral Lichen Planus: A Systematic Review and Meta-Analysis in the Context of the Risk in Oral Lichen Planus
Source: Dent J (Basel). 2026 Apr 8;14(4):217. doi: 10.3390/dj14040217 (PMC13114415; doi:10.3390/dj14040217)
Supplement: Supplementary file 1 [file dentistry-14-00217-s001.zip › Supplementary Table S1.pdf]

Supplementary Table S1. Table of Search Strategies in Databases

Data base	Search strategy<br>(search date: May, 2025)	Results
PubMed	("lichen planus"[MeSH Terms] OR "lichen planus") **AND** (skin[MeSH Terms] OR skin OR cutaneous OR cutaneal OR epidermal OR epidermic OR dermal OR dermic OR nail OR nails OR scalp OR genitalia [MeSH Terms] OR genitalia OR genital OR genitals OR vulva[MeSH Terms] OR vulva OR vulvas OR vulvar OR vulval OR vagina[MeSH Terms] OR vaginas OR vaginal OR vulvovaginal OR penis[MeSH Terms] OR penis OR penile OR extraoral) **AND** (neoplasms[MeSH Terms] OR neoplasms OR neoplasm OR cancer OR cancers OR carcinoma OR carcinomas OR neoplasia OR neoplasias OR malignant OR malignancy OR malignancies)	1,317
Scopus	TITLE-ABS-KEY("lichen planus") **AND** TITLE-ABS-KEY(skin OR cutaneous OR cutaneal OR epidermal OR epidermic OR dermal OR dermic OR nail OR nails OR scalp OR genitalia OR genital OR genitals OR vulva OR vulvas OR vulvar OR vulval OR vaginas OR vaginal OR vulvovaginal OR penis OR penile OR extraoral) **AND** TITLE-ABS-KEY(neoplasms OR neoplasm OR cancer OR cancers OR carcinoma OR carcinomas OR neoplasia OR neoplasias OR malignant OR malignancy OR malignancies)	2,338
Embase	("lichen planus") **AND** (skin OR cutaneous OR cutaneal OR epidermal OR epidermic OR dermal OR dermic OR nail OR nails OR scalp OR genitalia OR genital OR genitals OR vulva OR vulvas OR vulvar OR vulval OR vaginas OR vaginal OR vulvovaginal OR penis OR penile OR extraoral) **AND** (neoplasms OR neoplasms OR cancer OR cancers OR carcinoma OR carcinomas OR neoplasia OR neoplasias OR malignant OR malignancy OR malignancies)	509
Web of Science	TS=("lichen planus") **AND** TS=(skin OR cutaneous OR cutaneal OR epidermal OR epidermic OR dermal OR dermic OR nail OR nails OR scalp OR genitalia OR genital OR genitals OR vulva OR vulvas OR vulvar OR vulval OR vaginas OR vaginal OR vulvovaginal OR penis OR penile OR extraoral) **AND** TS=(neoplasms OR neoplasm OR cancer OR cancers OR carcinoma OR carcinomas OR neoplasia OR neoplasias OR malignant OR malignancy OR malignancies)	861
LILACS	("lichen planus" OR "líquen plano") **AND** (skin OR pele OR piel OR genitalia OR genitália OR genitales) **AND** (neoplasms OR neoplasias OR neoplasmas)	40
Google Scholar	First 100 more relevant hits. No patents and no citations.<br>("cutaneous lichen planus" OR "genital lichen planus") **AND** (cancer OR carcinoma OR malignant)	100
ProQuest	TI,AB("lichen planus") **AND** TI,AB(skin OR cutaneous OR cutaneal OR epidermal OR epidermic OR dermal OR dermic OR nail OR nails OR scalp OR genitalia OR genital OR genitals OR vulva OR vulvas OR vulvar OR vulval OR vaginas OR vaginal OR vulvovaginal OR penis OR penile OR extraoral) **AND** TI,AB(neoplasms OR neoplasm OR cancer OR cancers OR carcinoma OR carcinomas OR neoplasia OR neoplasias OR malignant OR malignancy OR malignancies)	291
