# Supplementary material for: Malignant Transformation in Extraoral Lichen Planus: A Systematic Review and Meta-Analysis in the Context of the Risk in Oral Lichen Planus
Source: Dent J (Basel). 2026 Apr 8;14(4):217. doi: 10.3390/dj14040217 (PMC13114415; doi:10.3390/dj14040217)
Supplement: Supplementary file 1 [file dentistry-14-00217-s001.zip › Supplementary Table S2.pdf]

Supplementary Table S2. Excluded articles and the reasons for their exclusion

Study (title, author and year)	Reasons for exclusion
Is vulvovaginal lichen planus associated with vaginal carcinoma? Alves et al., 2024	1
Characterising risk of progression to vulval malignancy in vulval lichen planus. Kherlopian and Fischer, 2021	3
Incidence of vaginal neoplasia with anogenital Lichen planus. Eberz and Regauer, 2009	3
Vulvar lichen planus and the risk of vulvar neoplasia. Lyra et al., 2019	3
Exploring the relationship between lichen planopilaris and skin cancer. Maazi et al., 2024	2
Oral and non-oral lichen planus show genetic heterogeneity and differential risk for autoimmune disease and oral cancer. Reeve et al., 2024	4
Vulvar malignancy in biopsy-proven vulval lichen planus: A retrospective review of 105 cases. Kherlopian and Fischer, 2020	2
Risk of vaginal neoplasia in anogenital lichen planus in women. Eberz and Regauer, 2009	3
Vulvar Lichen Planus: A Risk Factor for Vulvar High-Grade Squamous Intraepithelial Lesion Recurrence? Preti et al., 2018	2
Is Vulvovaginal Lichen Planus Associated With Squamous Cell Carcinoma? Day et al., 2018	5
Erosive vulvar lichen planus: a cohort at risk for cancer? Kennedy et al., 2008	3
Squamous cell carcinoma (SCC) and intraepithelial neoplasia among patients diagnosed with erosive lichen planus (LP): A study of 130 patients. Ghandi et al., 2023	2
Squamous cell carcinoma developed on inflammatory dermatoses followed at a tertiary hospital between the years 2000 and 2020. Magalhães et al., 2023	5
Vulvar Lichen Planus and Cancer. Selim, 2014	3
Concomitant hypertrophic lichen planus and squamous cell carcinoma: Clinical features and treatment outcomes. Idriss et al., 2022	1

Reason 1 = Case reports/case series
Reason 2 = Letter
Reason 3 = Full text unavailable.
Reason 4 = The article addressed only cases of oral lichen planus.
Reason 5 = Wrong population - It is not possible to differentiate the cases of lichen planus.
